# Literary Notes

**Published:** 1896-12

**Authors:** 


### Literary Notes.

It Pays to Take Your Home Medical Journal.—Many doctors have no idea of the number of inquiries which come to the local medical journal regarding the physicians living within the territory covered by such a periodical. The doctor hundreds of miles away from the home office of the journal may be as anxiously inquired about as the one living in the building where it is published. Life insurance companies, accident companies, bond and security companies, business houses and banks, corporations of all kinds and private individuals and firms write for information. The journal is looked upon as a sort of general bureau, which is drawn upon for information from all quarters. There is on the part of the journal a presumption that something is wrong with the medical man who does not take his home journal, and such *rara avis* needs to be very well known by the periodical to have its warmest endorsement. Again, if the doctor pays his subscriptions promptly, it helps to throw light upon the question so often asked about his financial standing.—*Medical Sentinel.*

---

MESSRS. WILLIAM WOOD & COMPANY, medical publishers, New York, have just published a photogravure entitled Anesthesia. It is not intended to be a correct representation of the modern scientific method of administering anesthetics; but the picture idealises in an artistic manner one of the greatest discoveries of modern times. It is one of the series of pictures designed for physicians' offices and libraries, and in our view is the most attractive of the group. It is an exact copy of the original India proof engraving, and is printed on extra heavy plate paper suitable for framing.

---

THE *Journal of Nervous and Mental Disease* announces the following arrangement of its staff for 1897: Editors, Dr. Chas L. Dana, Dr. F. X. Dercum, Dr. Philip Coombs Knapp, Dr. Chas. K. Mills, Dr. Jas. J. Putnam, Dr. B. Sachs, Dr. M. Allen Starr; associate editors, Dr. Philip Meirowitz, Dr. Wm. G. Spiller; managing editor, Dr. Chas. Henry Brown, 25 West 45th street, New York. This sterling magazine is a representative in its field and deserves the support of all neurologists. All editorial and business communications should be addressed to the managing editor.

